# Music and Exercise in Concert: Uncovering Synergistic Mechanisms for Enhancing Brain Health and Cognitive Resilience—A Narrative Review

**DOI:** 10.1002/brb3.71735

**Published:** 2026-08-30

**Authors:** Sanxia Yang, Qingluan Liu, Minghui Wang, Yidan Zhang

**Affiliations:** ^1^ College of Arts Wuhan Sports University Wuhan China; ^2^ Graduate School Wuhan Sports University Wuhan China

**Keywords:** cognition, dance, neuroinflammation, neural oscillations, neuroplasticity, non‐pharmacological intervention

## Abstract

**Aim:**

As the cornerstone of cognition, emotion, and social well‐being, brain health faces escalating threats from aging, neurodegenerative diseases, and sedentary lifestyles. Non‐pharmacological interventions have attracted growing interest owing to their favorable safety profiles and potential for widespread implementation. This review aims to examine the individual and synergistic effects of music and exercise on brain health.

**Methods:**

This narrative review integrates literature identified through searches of PubMed and Web of Science using terms related to music, exercise or physical activity, and brain health and offers a concept‐driven synthesis of the underlying neurobiological mechanisms and potential combined effects.

**Findings:**

Music modulates neurotransmitters and neural oscillations via reward and emotion networks, while exercise enhances neuroplasticity by improving energy metabolism, neurotrophic signaling, and cerebrovascular function. Their potential synergistic interaction may arise from music's capacity to temporally structure and modulate the neurochemical and metabolic states induced by exercise. Combined interventions may further enhance synaptic plasticity and network integration by mitigating neuroinflammation and oxidative stress. They may offer additional benefits for cognition, mood, and motor control beyond either intervention alone. Dance, a prototypical integration of music and movement, activates multisensory and social networks and has shown potential benefits in Alzheimer's disease, Parkinson's disease, depression, and autism.

**Conclusion:**

Music and exercise may engage convergent and potentially interacting mechanisms, including neuroplasticity, metabolic regulation, and neuroinflammatory modulation, forming an integrated framework for brain health. Dance exemplifies this music–movement integration by engaging multisensory and socio‐emotional networks, highlighting its translational potential for promoting brain function.

## Graphical abstract

Music and physical exercise share convergent neurobiological mechanisms, including neuroplasticity, metabolic regulation, and neuroinflammatory modulation. Integrating music with exercise produces synergistic effects that enhance brain function beyond single interventions. Dance exemplifies music–movement integration, promoting cognitive, emotional, and motor functions.

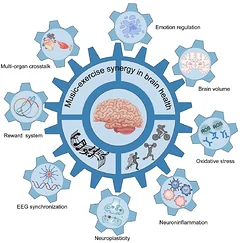

**Abbreviations**
AD: Alzheimer's disease
ADL: Activities of daily living
ASD: Autism spectrum disorder
BAIBA: β‐Aminoisobutyric acid
BDNF: Brain‐derived neurotrophic factor
BHB: β‐Hydroxybutyrate
CBF: Cerebral blood flow
EEG: Electroencephalogram
FGF21: Fibroblast growth factor 21
HPA: Hypothalamic‐pituitary‐adrenal
m6A: N6‐methyladenosine
MCI: Mild cognitive impairment
NF‐κB: Nuclear factor kappa‐B
PD: Parkinson's disease
PFC: Prefrontal cortex
PGC‐1α: Peroxisome proliferator‐activated receptor gamma coactivator 1‐alpha
SAM: S‐Adenosylmethionine
SCFAs: Short‐chain fatty acids
SIRT1: Sirtuin 1
TCA: Tricarboxylic acid
VEGF: Vascular endothelial growth factor

## 1 Introduction

With global population aging, the incidence of cognitive impairment, mood disorders, and neurodegenerative diseases is steadily increasing, posing serious threats to quality of life and societal well‐being (Livingston et al. 2020). Although pharmacological treatments for brain disorders continue to advance, their effectiveness, safety, and accessibility remain limited. Consequently, the development of safe, low‐cost, and widely applicable non‐pharmacological interventions has become a major research focus in neuroscience (Herholz et al. 2013). Contemporary digital environments may further challenge cognitive resilience, as persistent digital interruptions and media multitasking can fragment sustained attention and reduce opportunities for uninterrupted thinking (Dhahbi et al. 2026), underscoring the urgent need for accessible and effective non‐pharmacological strategies to support brain health.

Among various non‐pharmacological approaches, physical exercise, music‐based interventions, and dance have received particular attention for their feasibility and diverse benefits to brain health (Bhandarkar et al. 2024; Erickson et al. 2011; Gassner et al. 2022). Evidence indicates exercise improves cognitive function and emotional regulation by up‐regulating brain‐derived neurotrophic factor (BDNF), enhancing neural plasticity, and strengthening brain network function and metabolic efficiency (Wang et al. 2024). Similarly, music can activate the prefrontal cortex, limbic system, and reward pathways; upregulate neurotransmitters such as dopamine and oxytocin; and enhance mood, memory, and attention (Speranza et al. 2022). Beyond passive listening and instrumental music training, singing has emerged as an accessible form of active music‐based intervention for older adults. Active singing has been associated with improved cognitive function and greater life satisfaction among individuals with dementia (Maguire et al. 2015). Singing engages auditory perception, vocal–motor control, language processing, memory retrieval, and emotional expression and may promote neuroplasticity by strengthening auditory–motor integration and supporting white matter integrity (Tragantzopoulou and Giannouli 2025).

An increasing number of studies have explored the potential of combining exercise with music interventions. For instance, integrating music therapy into family‐based exercise programs has been shown to foster a more emotionally supportive rehabilitation environment, thereby reducing depression risk in stroke patients (Palumbo et al. 2022). Moreover, in conditions such as cancer, traumatic brain injury, insomnia, and dementia, music interventions alleviate disease‐related anxiety, depression, and stress responses (Cho et al. 2023; Du et al. 2024; Fu et al. 2023; Jespersen et al. 2022; Moschonas et al. 2023). These psychological symptoms are critical determinants of the effectiveness of exercise interventions. In this context, music may act as a contextual facilitator, amplifying the positive effects of exercise on brain function (Tabei et al. 2023).

Although the benefits of exercise and music interventions are well documented, systematic reviews and integrative analyses addressing their neural overlap and synergistic effects remain limited. For neurological disorders such as cognitive impairment, depression, and Parkinson's disease (PD), it remains unclear whether combined interventions are superior to single‐modality approaches or whether their mechanisms interact.

Therefore, leveraging recent advances in neuroscience, this article examines independent effects and potential synergistic mechanisms of music and physical exercise on brain health, with particular attention to pathways involving neuroplasticity, neurotransmitter regulation, energy metabolism, brain network function, and neuroinflammation. Furthermore, by examining dance, a prototypical music–exercise fusion intervention, we discuss its potential applications in brain health and outline key questions and future research directions.

## 2 Music and Brain Function

As a multidimensional sensory stimulus deeply rooted in human culture, music evokes emotions, memories, and aesthetic experiences. Previous studies indicate that music processing engages multiple brain regions, including, but not limited to, the amygdala, nucleus accumbens, hippocampus, insula, cingulate gyrus, and orbitofrontal cortex (Koelsch 2014). Neuroimaging studies report that music‐based neurorehabilitation enhances structural connectivity and executive function in the prefrontal cortex and right inferior frontal gyrus while promoting gray matter plasticity and neural integration (Martínez‐Molina et al. 2022; Siponkoski et al. 2020). In neonatal intensive care, music interventions demonstrate neuroprotective effects by optimizing acoustic stimulation and promoting brain microstructural development in extremely premature infants (Dewan et al. 2024). Moreover, passive music listening has been increasingly investigated as a simple, low‐cost, and easily implemented approach to supporting cognitive function in middle‐aged and older adults. Recent evidence suggests that brief exposure to Mozart's music may improve visuospatial working memory in both young and older adults. These effects may involve emotional activation and central executive attentional processes (Giannouli et al. 2024). However, the cognitive effects of passive music listening are inconsistent across studies. They may vary with the characteristics of the musical excerpt, task demands, individual music preferences, and prior musical experience. For example, listening to Vivaldi's music may selectively improve verbal fluency, whereas exposure to music by Mozart or Beethoven has not consistently improved performance on reverse‐order memory tasks (Giannouli et al. 2018; Giannouli et al. 2010).

From a neural perspective, the core components of music are melody, harmony, and rhythm. Although distinct neural networks process these elements, they interact to create a unified musical experience (Koelsch 2011; Zatorre et al. 2007). Melody refers to the temporal pattern of pitch. Its processing mainly engages the auditory cortex, prefrontal cortex, and superior temporal gyrus and is closely linked to tonal expectations and emotional resonance (Wang et al. 2014; Koelsch 2014). Harmony consists of multiple pitches presented simultaneously. Its recognition engages prefrontal and auditory association cortices, which contribute to the processing of musical grammar and harmony and modulate emotional responses (Pallesen et al. 2005; Pyott et al. 2024). Rhythm, the temporal structure of music, is processed by both auditory and motor systems. Studies show that even without explicit beat cues, metronome stimulation leads the brain to mark certain beats as more prominent, indicating that rhythm perception is inherently predictive and automatic (Brochard et al. 2003).

Recent neuroscience research demonstrates that music perception depends on the auditory system and is closely tied to cognitive processes such as movement, emotion, and learning, prompting a re‐examination of its neural mechanisms (Vuust et al. 2022). The predictive coding model has emerged as a key framework for understanding music perception. According to this model, the brain does not passively receive musical stimuli but generates predictions based on prior experience, actively infers upcoming melody, rhythm, or harmony, and continuously updates predictions by comparing them with incoming input. In other words, music processing is a dynamic cycle of perception, prediction, and error correction. This mechanism applies not only to sound structures such as melody and rhythm but also to cognitive domains including emotion, attention, learning, and memory (Vuust et al. 2022). For example, pulse and beat are not always explicitly present in sound input but are inferred through the brain's hierarchical predictive processing of rhythm (Large and Kolen 1994; Vuust and Witek 2014). Consistent with this predictive framework, the early emergence of beat perception during infancy and childhood is instrumental in shaping rhythmic memory, thereby facilitating subsequent music learning and language acquisition (Cutietta and Booth 1996; Palmer and Krumhansl 1990). Moreover, music information is encoded into semantic or structural units across multiple levels. The prediction mechanism activates neural networks in the prefrontal, temporal, and parietal lobes, thereby integrating features, mobilizing associations, strengthening memory, and enhancing emotional resonance and memory retrieval (Ren and Brown 2024).

The brain's predictive engagement with music is physically manifested in the synchronization of neural oscillations, as described by the neural resonance theory, elucidating the dynamic coupling between music and neural oscillations. The theory proposes that music strengthens neural network stability and flexibility by synchronizing neural oscillations. This synchronization positively influences emotional regulation, interpersonal coordination, and social connectedness (Harding et al. 2025). Electroencephalogram (EEG) studies demonstrate that music listening significantly increases synchronization between delta and theta waves, indicating that music enhances attention allocation and executive control by modulating neural oscillatory rhythm (Meltzer et al. 2015; Nandi et al. 2023; Tian et al. 2025). Long‐term music training not only enhances neural adaptability to rhythmic synchronization but also induces profound structural and functional plasticity in the brain, thereby improving cognitive flexibility and learning efficiency (Malekmohammadi et al. 2023; Yurgil et al. 2020). Auditory stimulation activates motor‐related networks, generating pulse frequency oscillations in the motor planning system. Although not fully aligned with the surface frequency of musical rhythm, these oscillations may underlie the neural construction of rhythmic expectations (Large et al. 2015). Theta wave activity is a key marker of music‐induced neuroplasticity, continuously influencing neural encoding, memory consolidation, and information retrieval (Bugos et al. 2022).

More importantly, music robustly activates the dopaminergic reward system, thereby promoting pleasure and motivation. Studies indicate that music‐induced reward responses exhibit distinct predictive properties. When music meets or violates listeners’ expectations, the reward system is activated at different stages, producing varying levels of pleasure (Salimpoor et al. 2011; Salimpoor et al. 2015). Furthermore, music‐induced dopamine release enhances emotional experience and facilitates memory encoding and consolidation, with effects particularly pronounced in individuals with high reward sensitivity (Ferreri and Rodriguez‐Fornells 2017). Beyond dopamine, oxytocin and testosterone are also implicated in the neural regulation of music‐induced social trust, intimacy, and learning motivation, thereby extending the cognitive boundaries of music's impact on brain function (Ferreri et al. 2021; Fukui and Toyoshima 2023; Harvey 2020).

Therefore, music comprehensively promotes emotion regulation, cognitive processing, and social behavior control by mobilizing activities in multiple brain regions and integrating prediction mechanisms, neural oscillations, and reward pathways, thus forming an important neural basis for improving brain function, as we will explore later, establishing a favorable neural foundation for potential synergy with exercise.

## 3 Exercise and Brain Function

As a widely applied non‐pharmacological intervention, exercise enhances cognitive function and mood and may help slow cognitive decline associated with neurodegenerative disorders (Ben Ezzdine et al. 2025; Schootemeijer et al. 2020; Valenzuela et al. 2020). Recent evidence further suggests that these benefits may be partly mediated by exercise‐induced neuroplasticity, including enhanced neurotrophic signaling, synaptic connectivity, and neurogenesis, as well as reduced neuroinflammation and oxidative stress (Ben Ezzdine et al. 2025). Collectively, these effects are mediated through a hierarchical physiological cascade that begins with acute metabolic remodeling, extends to neurochemical and vascular adaptations, and culminates in systemic cross‐organ communication and epigenetic regulation. A key manifestation of this cascade is the release of exercise‐induced endogenous metabolites. These small molecules cross the blood‐brain barrier to regulate neuronal metabolism, signal pathways, and inflammatory status, thereby serving as key mediators between exercise and brain function (El Hayek et al. 2019; Li et al. 2022). This multi‐level framework integrates core mechanisms, including increasing cerebral blood flow, regulating neurotransmitter release, improving energy metabolism, and enhancing neuroplasticity (Key and Szabo‐Reed 2023; Lenze et al. 2022). In recent years, increasing attention has been directed toward exercise‐induced endogenous metabolites. The most immediate effect of exercise is a systemic metabolic shift that produces a class of signaling molecules that directly support brain health. Among these, lactate, ketone bodies, and kynurenine are representative exercise metabolites under intensive investigation. Importantly, exercise‐induced metabolite profiles vary by exercise modality: high‐intensity aerobic and anaerobic exercise tend to favor lactate production, whereas prolonged endurance exercise is more closely associated with ketone‐body availability, and endurance exercise may also influence β‐aminoisobutyric acid (BAIBA) and kynurenine metabolism (Wang et al. 2024). Lactate, once dismissed as a mere byproduct of anaerobic glycolysis, is now recognized as a key signaling molecule with pleiotropic neuroactive properties. Lactate not only can cross the blood–brain barrier to provide neuronal energy substrates, but also can activate the sirtuin 1 (SIRT1) signaling pathway, promote hippocampal BDNF expression, and enhance neuroplasticity and memory formation (El Hayek et al. 2019). Additionally, lactate also can regulate neuronal excitability via hydroxycarboxylic acid receptor 1 (HCA1/GPR81) receptors, thereby contributing to cognitive processing (El Hayek et al. 2019; Lauritzen et al. 2014). Ketone bodies, particularly β‐hydroxybutyrate, serve as key energy substrates during prolonged endurance exercise or low‐carbohydrate diets. Beyond their metabolic role, they exert epigenetic effects by inhibiting histone deacetylases, enhancing synaptic plasticity and antioxidant capacity, thereby improving cognition and delaying neurodegeneration (López‐Ojeda and Hurley 2023). As an intermediate in the tryptophan pathway, kynurenine is converted into the neuroprotective metabolite kynurenic acid via peroxisome proliferator‐activated receptor gamma coactivator 1‐alpha (PGC‐1α) induction, thereby reducing neuroinflammation and enhancing stress resilience (Agudelo et al. 2014).

While these metabolites act directly on neurons, exercise also systematically optimizes the brain's logistical support system, thereby creating an ideal environment for the aforementioned effects. This process begins with a comprehensive remodeling of the cerebrovascular system and the neurochemical environment. First, exercise significantly improves cerebrovascular function, including oxygen delivery and nutrient transport in the brain. Exercise upregulates monocarboxylate and glucose transporters, facilitating the transport of lactate and ketone bodies across the blood–brain barrier and their neuronal utilization (Pervaiz et al. 2023; Takimoto and Hamada 2014). In addition, exercise increases brain capillary density and enhances blood‐brain barrier function, thereby improving metabolite accessibility and utilization efficiency in the brain (Małkiewicz et al. 2019; Wang et al. 2023). Simultaneously, exercise efficiently modulates the neurotransmitter system. It markedly increases the synthesis and release of key neurotransmitters, including dopamine, serotonin, and norepinephrine. Dopamine is critically involved in reward, motivation, and motor control. Exercise modulates dopaminergic signaling within the reward system and may contribute to improved motor performance in patients with PD (Fisher et al. 2013; Sacheli et al. 2019). Elevated serotonin contributes to mood regulation and depression alleviation, representing a key mechanism for mitigating psychological stress upon exercise (Chaouloff 1997; Meeusen et al. 2007).

The benefits of exercise extend well beyond the brain itself; it engages entire organ systems and triggers profound epigenetic modifications to achieve long‐term regulation of brain health at the whole‐organism level. Specifically, exercise‐induced metabolites act directly in the brain and coordinately regulate brain health through multiple organ‐brain axes. For example, BAIBA, secreted by skeletal muscle, regulates hepatic fatty acid oxidation, promotes white fat browning, and exerts anti‐inflammatory and energy‐regulatory effects in the brain (Colaianni et al. 2020; Roberts et al. 2014). Gut microbial short‐chain fatty acids (SCFAs), such as butyrate, cross the blood–brain barrier to regulate nerve growth factor expression, synaptic plasticity, and inflammatory responses (Clarke et al. 2014; Dehghani et al. 2023). In addition, exercise‐induced regulation of hepatic S‐adenosylmethionine (SAM) synthesis may enhance central nervous system function and synaptic plasticity by restoring N6‐methyladenosine (m6A) methylation (Yan et al. 2022). These multi‐organ interactions highlight the integrative role of exercise metabolites in the muscle‐brain, gut‐brain, adipose‐brain, and liver‐brain axes. A second line of evidence suggests that exercise metabolites contribute to long‐term neuroadaptation by modulating the epigenetic state of the nervous system. For instance, β‐hydroxybutyrate regulates genes involved in energy metabolism and neurotrophic signaling through histone β‐hydroxybutyrylation (Chen et al. 2017; Sleiman et al. 2016). Moreover, lactate‐induced protein lactylation is thought to activate genes critical for neural development and stress adaptation, thereby impacting memory and cognition (Bozzo et al. 2013; Galle et al. 2022).

Importantly, the multi‐level benefits described above are differentially modulated by exercise modality. Different exercise modalities elicit distinct metabolic responses, leading to varied effects on brain function. Although all exercise modalities involve overlapping contributions from oxidative and non‐oxidative energy systems, low‐ to moderate‐intensity aerobic exercise generally places greater demands on oxidative metabolism, thereby supporting mitochondrial function, fatty acid oxidation, and sustained energy supply (Hargreaves and Spriet 2020). Long‐term aerobic exercise improves brain oxygenation and insulin sensitivity, enhances the utilization of fatty acids and ketone bodies, and exerts neuroprotective effects in conditions such as cognitive decline and Alzheimer's disease (AD) (Cox et al. 2016; Escribano et al. 2005; Gaitán et al. 2021; Liubaoerjijin et al. 2016; Valenzuela et al. 2020). Traditional resistance exercise tends to rely more on phosphagen and glycolytic pathways. However, the relative contribution of each energy system varies with exercise load, repetition number, movement velocity, and rest intervals (Mitchell et al. 2024). High‐intensity interval training differs from traditional resistance exercise because its repeated high‐intensity bouts involve substantial anaerobic contributions, while oxidative metabolism remains important during recovery and across successive intervals (Buchheit and Laursen 2013; Laursen and Jenkins 2002). These modality‐specific metabolic demands may differentially influence stress responses, neurotransmitter release, and reward‐related mechanisms (Chaouloff 1997; Huang et al. 2018; Li et al. 2022; Nasb et al. 2024). Moreover, these training modalities improve metabolic adaptability by enhancing lactate clearance, fatigue resistance, and mood stability (Laursen and Jenkins 2002). Notably, the physiological responses elicited by different exercise modalities may translate into distinct cognitive outcomes. Moderate‐intensity aerobic exercise may benefit memory, executive function, and mood regulation, whereas moderate‐to‐high‐intensity resistance training may be particularly beneficial for visuospatial processing and executive function. However, findings regarding concurrent aerobic and resistance training remain mixed, suggesting that its cognitive benefits may depend on exercise modality, intensity, and program design (Dhahbi et al. 2025). Beyond exercise modality and intensity, psychological responses may also depend on training structure and task constraints. For example, manipulating bout duration and player number during soccer small‐sided games influenced mood‐state profiles, with continuous 12‐min bouts associated with lower total mood disturbance than shorter intermittent formats (Farhani et al. 2025). Furthermore, exercise timing also influences metabolic regulatory efficiency. Under circadian regulation, exercise performed during physiologically active periods significantly enhances fatty acid oxidation, glucose metabolism, and tricarboxylic acid (TCA) cycle activity, thereby improving systemic metabolic efficiency (Sato et al. 2019; Sato et al. 2022). These findings suggest that optimizing exercise timing may maximize brain activation and provide theoretical support for personalized precision exercise interventions.

In summary, exercise enhances brain health through a well‐defined hierarchical network: it directly regulates neuronal function via metabolites, provides robust support by optimizing the cerebrovascular and neurochemical environment, and ultimately achieves systemic optimization and long‐term programming from physiological to genetic levels by coordinating multi‐organ crosstalk and epigenetic mechanisms. This comprehensive framework not only elucidates the principles by which exercise promotes brain health but also clearly reveals that the favorable intracerebral environment—characterized by enhanced energy metabolism, plasticity, and systemic coordination—created by exercise establishes an ideal physiological foundation for synergistic effects with other interventions, such as music.

**FIGURE 1:**
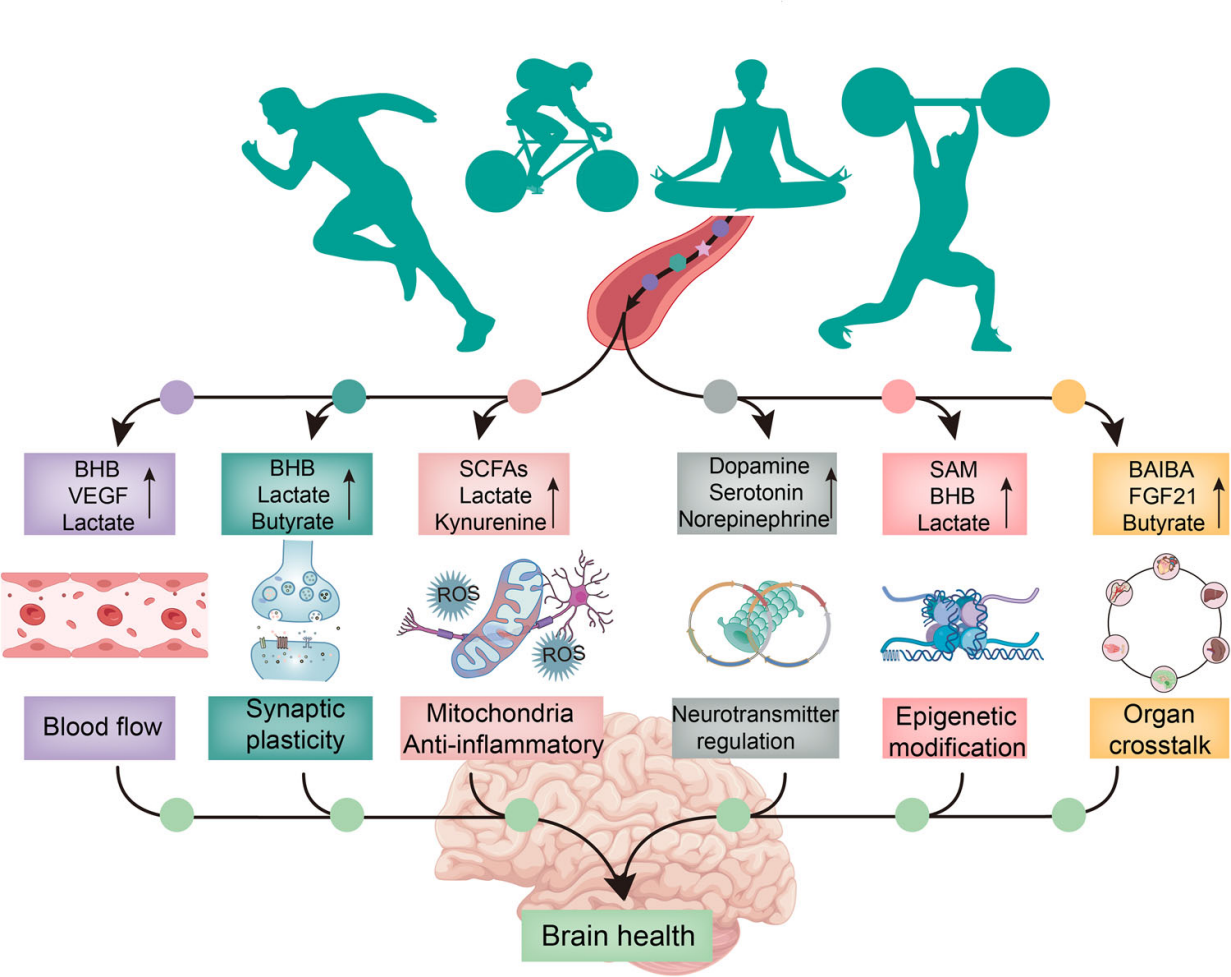
Key exercise‐induced metabolites and their roles in promoting brain health. Abbreviations: BAIBA, β‐aminoisobutyric acid; BDNF, brain‐derived neurotrophic factor; BHB, β‐hydroxybutyrate; FGF21, fibroblast growth factor 21; SAM, S‐adenosylmethionine; SCFAs, short‐chain fatty acids; VEGF, vascular endothelial growth factor.

Exercise promotes the secretion of metabolites and signaling molecules such as lactate, β‐hydroxybutyrate, vascular endothelial growth factor (VEGF), butyrate, kynurenine, fatty acids, SAM, BAIBA, fibroblast growth factor 21 (FGF21), and various neurotransmitters. These mediators enhance cerebral blood flow, synaptic plasticity, and mitochondrial function, regulate inflammatory responses and epigenetic modifications, and facilitate multi‐organ interactions, thereby collectively supporting brain health (Figure 1).

## 4 Convergent and Interactive Mechanisms Underlying Music and Exercise Interventions

As two widely adopted non‐pharmacological interventions, music and exercise both significantly enhance brain neuroplasticity. For example, long‐term music training upregulates plasma BDNF and modulates neurotransmitters such as serotonin, thereby enhancing synaptic connectivity and cognitive flexibility (Park et al. 2023; Sun et al. 2024; Xue et al. 2023; Zhou et al. 2025). Regular aerobic exercise promotes hippocampal neurogenesis, increases synaptic protein expression, and improves learning and memory by regulating hormones such as dopamine and melatonin (Di Liegro et al. 2019; Jiang et al. 2024; Terao and Kodama 2024). Clinically, exercise combined with music has been associated with the preservation of prefrontal gray matter and improvements in executive and spatial cognition, suggesting potential additional benefits for healthy brain aging (Tabei et al. 2017). In addition, music training is regarded as an important source of cognitive reserve (Wolff et al. 2023). In patients with mild cognitive impairment and dementia, multimodal interventions integrating music, games, and exercise show greater cognitive benefits than exercise alone (Voinescu et al. 2024). Related evidence across children, adolescents, and healthy or clinical populations further supports the broader roles of music‐ and movement‐based interventions in cognitive and emotional function (Bentley et al. 2023; Fan et al. 2024; Gustavson et al. 2023; Lin et al. 2024), underscoring the complementary roles of music and exercise in neurotrophic regulation. This section examines these convergent mechanisms across neurochemical, oscillatory, and neuroprotective domains and considers how their interactions may contribute to the potential complementary effects of combined interventions.

At the neurotransmitter level, both music and exercise engage the dopaminergic reward system, thereby supporting motivation, pleasure, and emotional regulation. Listening to or performing music activates the nucleus accumbens and caudate nucleus, inducing dopamine release, with effects closely tied to subjective pleasure (Ferreri et al. 2019). Aerobic exercise may alleviate depression and motivational deficits by enhancing dopaminergic signaling within the reward system (Chen et al. 2019). Exercise has been reported to increase perceived musical pleasure, suggesting that exercise‐related changes in arousal and affect may modulate music reward (Hove et al. 2021). In addition, the rewarding effects of exercise enhance individual initiative, while variations in gut microbiota may partly explain interindividual differences in exercise performance (Dohnalová et al. 2022). Music exposure also modulates the gut microbiome and enhances immune function (Zhu et al. 2025), underscoring its role in promoting exercise adherence and health benefits. Beyond dopamine, music and exercise can both promote the release of serotonin and oxytocin, thereby contributing to emotional stability and social connectedness (Evers and Suhr 2000; Kumar et al. 1999; Speranza et al. 2022). In addition, glutamate and norepinephrine are also implicated in related neural regulatory processes (Mao et al. 2022; Xu et al. 2008). Therefore, music not only motivates exercise initiation but also enhances the cognitive and psychological benefits of exercise by activating emotion‐ and motivation‐related neural pathways. Consistently, Kinect‐based exercise therapy combined with music art improves neurological function, sleep quality, and mood in patients with AD (Hallett and Lamont 2019; Li and Li 2024).

Regarding the synchronization of EEG signals, music and exercise interventions enhance alpha‐ and theta‐band synchronization, thereby strengthening cross‐regional networks that support cognitive flexibility, attention control, and emotion regulation (Cheung et al. 2018; Hobeika et al. 2022; Shimizu et al. 2018; Thach et al. 2025; Wang et al. 2024). Research shows that during familiarization, alpha/theta phase synchronization in frontal, temporal, and parietal regions is significantly enhanced, facilitating emotional processing and information integration (Malekmohammadi and Cheng 2024). Evidence from music perception studies further indicates that musical expertise may modulate EEG phase synchrony during music listening. Compared with non‐musicians, musicians show stronger distributed cortical phase synchrony, particularly in the gamma band. This pattern suggests that long‐term musical training may shape context‐dependent functional connectivity during music processing (Bhattacharya and Petsche 2005). Exercise interventions also enhance functional connectivity among regions involved in emotion and perception during extreme or acute exercise (Ashtari et al. 2022; Ciria et al. 2019; Menicucci et al. 2016). More importantly, when music was combined with exercise, frontal theta synchronization was further enhanced during tasks, potentially reflecting music's amplifying effect on attention allocation and neuroplasticity regulation (Balasubramanian et al. 2024). In addition, combining culturally specific music (e.g., gamelan) with sensorimotor rhythm training has been shown to improve EEG synchronization and language processing in children with autism (Antara et al. 2025), suggesting potential value for combined interventions in special populations. In addition, combining 15‐Hz beta‐frequency binaural beats with preferred music was associated with lower perceived exertion and more positive affective responses during inter‐round recovery in amateur kickboxers, suggesting that frequency‐specific auditory stimulation may provide an additional approach to modulating the effects of music during exercise (Jebabli et al. 2025).

Regarding neuroprotection, music and exercise may converge on pathways involved in oxidative stress and inflammatory regulation. Aerobic exercise enhances the activity of antioxidant enzymes such as superoxide dismutase and catalase, reduces oxidative products such as malondialdehyde, and maintains immune homeostasis by downregulating pro‐inflammatory factors and upregulating anti‐inflammatory factors (Cho and Roh 2023). Music interventions also exhibit anti‐inflammatory and antioxidant potential by inhibiting nuclear factor kappa‐B (NF‐κB) signaling, activating the Nrf2 signaling pathway, and alleviating organ damage induced by stress or noise (Wang et al. 2024a; Wang H et al. 2024b). In animal studies, music exerts neuroprotective effects in postpartum depression mouse models by regulating synaptic plasticity and inflammatory responses (Fu et al. 2025a). Taken together, preclinical evidence suggests that music and exercise can each modulate inflammatory and oxidative‐stress pathways, indicating potentially convergent neuroprotective mechanisms (Gao et al. 2023; Lin et al. 2025; Rezaei Moghaddam et al. 2024; Wang M et al. 2023; Zhang et al. 2023). Preclinical studies suggest that the Toll‐like receptor 4 signaling pathway may represent a candidate mechanism shared by both interventions (Jin et al. 2025; Zhang et al. 2025). However, the relevance of these findings to human neuroinflammation and clinical interventions remains to be established. In addition, some studies suggest that, under specific conditions, music may aggravate inflammation and oxidative stress (Xiao et al. 2025). More broadly, intervention context and dosage must be carefully considered in clinical applications to optimize neuroprotective effects.

In summary, music and exercise engage convergent and potentially interacting pathways involving neurotrophic factors, neurotransmitter regulation, neural oscillations, and inflammatory and oxidative‐stress responses. These shared mechanisms provide a neurobiological rationale for investigating their combined application.

## 5 Potential Synergistic Effects: Evidence and Hypotheses

Building on the convergent neurobiological mechanisms outlined above, this section examines the empirical evidence for combined music–exercise interventions. Here, “synergistic” is used to describe potentially complementary or interacting effects across multiple pathways, rather than statistically demonstrated super‐additivity. We further consider how music‐related perceptual and attentional processes, individual music preferences, and intervention dosage may shape their effects. EEG and neuroimaging studies suggest that combining music and exercise may enhance alpha‐ and theta‐band synchronization, strengthen functional connectivity between frontal and motor regions, and support executive function and attentional control (Mom et al. 2023; Suwabe et al. 2021). In older adults, combined music–exercise interventions have also been associated with improvements in cognitive function and emotional well‐being, as well as the preservation of gray matter in relevant brain regions (Tabei et al. 2017). Recent meta‐analytic evidence further suggests that combined music and exercise therapy may reduce depressive symptoms (Luo et al. 2026). These findings suggest that music and exercise may exert complementary effects on brain structure and function through partially overlapping neural mechanisms.

Beyond amplifying shared mechanisms, music uniquely modulates perception and attention during exercise. Music inhibits fatigue‐related signals from reaching conscious processing by reducing cortical theta activity, limiting information exchange between somatosensory areas, and activating executive‐related regions such as the left inferior frontal gyrus, thereby improving concentration and task performance (Karageorghis et al. 2018).

This mechanism helps individuals maintain positive emotions and sustain attention during high‐intensity exercise, thereby enhancing performance in real‐world contexts. Music combined with exercise activates the brain–body interaction network through multimodal input, strengthens coupling between the brainstem arousal system and higher cognitive areas, and improves prefrontal gray matter volume, executive function, and language processing (Siponkoski et al. 2020). In low‐intensity exercise, music exerts an emotional separation effect by reallocating attention away from somatosensory signals, reducing fatigue, and enhancing the pleasure and sustainability of exercise (Bigliassi et al. 2018). Collectively, these findings may be interpreted within a proposed resource‐optimization framework. According to this framework, music may externally structure temporal patterns, regulate affective states, and redirect attention away from fatigue‐related signals, thereby facilitating more efficient allocation of attentional and motivational resources during exercise. Such resource reallocation may support motor performance, exercise adherence, and the overall exercise experience. However, this framework remains conceptual and has not yet been directly tested against a simple additive model. Notably, the efficacy of combined interventions is critically dependent not only on the exercise modality but also on individual music preference and rhythmic complexity: highly preferred or strongly rhythmic music is more likely to elicit positive emotions and autonomous movement, thereby reinforcing a positive cycle (Ballmann 2021; Suwabe and Kawase 2025), while recent meta‐analytic evidence in combat sports suggests that combining music with other ergogenic strategies may yield larger performance effects than music alone (Jebabli et al. 2026). A randomized crossover trial further supports this view. Self‐selected motivational music during warm‐up improved countermovement jump, medicine‐ball throw, sprint, and agility performance across different time of a day, with the greatest benefits observed during morning sessions (Bougrine et al. 2025). These findings suggest that personalized music selection may improve exercise performance by enhancing arousal and psychomotor readiness, particularly when physiological readiness is relatively low. Evidence from passive music listening is broadly consistent with this interpretation, suggesting that music may influence cognitive performance through emotional arousal, attentional allocation, and working memory‐related processes (Giannouli et al. 2024).

In addition, music‐evoked autobiographical memories hold unique value for cognitive recovery, particularly in patients with AD and other neurodegenerative disorders, effectively eliciting specific emotions and contextual memories (Kaiser and Berntsen 2023). However, the dosage of music intervention also requires careful consideration. When music volume exceeds 60 decibels, it may act as a noise stimulus, aggravating emotional dysregulation and memory impairment (Belojević 2024; Nian et al. 2023). In animal studies, music stimulation reduced hypothalamic‐pituitary‐adrenal (HPA) axis activity, thereby alleviating transport stress‐induced inflammation. In contrast, noise increased HPA activity, leading to more severe intestinal damage and oxidative stress (Fu et al. 2025b). Therefore, combining music and exercise may engage complementary neuromodulatory processes involving perceptual regulation, memory evocation, affective modulation, and dose‐dependent responses. However, current evidence does not establish whether these combined effects are additive or genuinely super‐additive. The reported effects of combined music–exercise interventions are summarized in Table 1.

**TABLE 1:** Combinatorial effects of music and exercise on brain health.

Participants	Study design	Exercise intervention	Music intervention	Key findings	Refs
Healthy elderly (*n =* 114)	Non‐randomized controlled trial	Online physical exercise (60 min/session; once weekly; 6 months, 20 sessions)	Synthesizer‐heavy dance‐pop music	Working memory ↑	(Tabei et al. 2023)
Healthy runner (*n =* 18)	Repeated‐measures study	Aerobic exercise (3 times)	10 favorite music clips	EEG delta activity ↑	(Schneider et al. 2010)
Chronic stroke patients (*n =* 25)	Randomized controlled trial	home exercise program (45 min/time; 2 times/week; 6 weeks)	Live interactive music creation	Depression ↓; BDNF, happiness ↑	(Palumbo et al. 2022)
Healthy young men (*n =* 9)	Crossover trial	Resistance exercise	Listen to music before exercise	Neuromuscular fatigue →	(Diehl et al. 2023)
Chronic stroke patients (*n =* 40)	Randomized controlled trial	eMST (60 min/times; 4 times/week; 10 weeks)	Rhythm music	Motor performance and executive function ↑	(Segura et al. 2024)
Middle‐aged and elderly women (*n =* 67)	Randomized controlled trial	Aerobic exercise (50 min/time; 3 times/week;12 weeks)	Rhythm music	BDNF ↑; Depression and fatigue ↓	(Yeh et al. 2015)
Healthy elderly (*n =* 144)	Non‐randomized controlled trial	Aerobic exercise (60 min/time; 1 time/ week; 48 weeks)	Dance pop	Frontal lobe gray and white matter volume ↑	(Tabei et al. 2017)
Healthy adults (*n =* 19)	Crossover trial	Grip strength exercise (30 trials)	Rhythm music	Brain fatigue ↓; Brain region activation ↑	(Bigliassi et al. 2018)
Patients with schizophrenia and depression (*n =* 84)	Randomized controlled trial	Aerobic exercise (60 min/time; 3 times/week, 12 weeks)	Patient's favorite music	Depression ↓	(Khanmohammadi et al. 2025)
PD patients (*n =* 50)	Randomized controlled trial	Aerobic exercise (30 min/session; 5 sessions/week; 8 weeks)	Rhythm music	PD symptoms ↓	(Calabrò et al. 2019)
Children with ASD (*n =* 13)	Repeated‐measures study	Aerobic exercise (20 min/time; 6 days)	Rhythm music	Exercise intensity ↑	(Woodman et al. 2018)
Healthy young people (*n =* 33)	Crossover trial	Aerobic exercise (10 min)	Rhythm music	Positive emotion and energy ↑	(Suwabe et al. 2021)
Healthy adults (*n =* 19)	Crossover trial	Jymmin (10 min)	Synthesizer music	Pain tolerance ↑	(Fritz et al. 2017)
Healthy adults (*n =* 41)	Crossover trial	Aerobic exercise (3 min)	Drum music	Executive function and Pleasure ↑	(Fukuie et al. 2023)
Patients with chronic pain (*n =* 24)	Crossover trial	Acute exercise (20 min)	Synthesizer music	Pain →; Anxiety ↓	(Schneider et al. 2022b)
Healthy adults (*n =* 42)	Crossover trial	Tai Chi, Ba Duan Jin (15 min/session)	Five elements music	Reaction time ↓; Emotion score ↑	(Zhao et al. 2025)
Older women with MCI (*n =* 13)	Repeated‐measures study	Aerobic exercise (60 min/time; 3 times/week; 12 weeks)	Rhythm music	Memory and executive function ↑; Depression ↓	(Domínguez‐Chávez et al. 2022)
People with dementia (*n =* 27)	Crossover trial	Jymmin (6 min; 5 times/week; 2 weeks)	Thera band music	Memory ↑	(Strong et al. 2022)
MCI patients (*n =* 39)	Randomized controlled trial	Multitasking movement music therapy (60 min/time; 1 time/week; 12 weeks)	Japanese traditional songs	Cognitive function, PFC blood flow, Functional connectivity ↑	(Shimizu et al. 2018)
Students with mental fatigue (*n =* 20)	Crossover trial	Moderate‐intensity aerobic exercise (20 min)	Fast‐paced classical music	Working memory and alertness ↑	(Dong et al. 2025)
Patients with brain injury (*n =* 35)	Randomized controlled trial	Rehabilitation exercise (30 min/session; 3 times/week; 4 weeks)	Jymmin music	Cognitive function and ADL ↑; mood →	(Schrader et al. 2025)
Healthy adults (*n =* 65)	Crossover trial	High‐intensity treadmill running (7 min)	Asynchronous motivational music	Positive affect ↑; prefrontal activation modulated	(Quan et al. 2026)
Male athletes (*n =* 34)	Crossover trial	Incremental cycling exercise to exhaustion	Motivational instrumental music	Endurance performance and brain network efficiency ↑	(Fan et al. 2026)
Amateur kickboxers (*n =* 19)	Randomized crossover trial	Simulated kickboxing combat	Preferred music and 15‐Hz binaural beats	Positive affect ↑; perceived exertion ↓	(Jebabli et al. 2025)
Healthy adults (*n =* 22)	Crossover trial	Light‐intensity aerobic exercise (10 min)	Rock music	CBF and executive function ↑	(Ayaz et al. 2025)

*Note*: ↑ indicates improvement/increase; ↓ indicates reduction; →, no significant change. Abbreviations: ADL, activities of daily living; ASD, autism spectrum disorder; BDNF, brain‐derived neurotrophic factor; CBF, cerebral blood flow; EEG, electroencephalogram; MCI, mild cognitive impairment; PD, Parkinson's disease; PFC, prefrontal cortex.

## 6 Dance as an Integrated Intervention for Brain Health

As an ecologically relevant form of music–movement integration, dance provides a useful model for examining the potential combined effects of music and physical activity. Unlike simple exercise or passive listening, dance requires the continuous integration of rhythm, movement, emotion, and social interaction, thereby representing a complex multimodal intervention with potential relevance to brain health. However, because dance also involves choreographic learning, cognitive demands, social interaction, and variable cardiovascular load, its observed effects cannot be attributed exclusively to the interaction between music and exercise.

Dance has been associated with structural and functional neuroplastic changes across multiple brain regions. It activates neural networks through multisensory input, thereby enhancing brain plasticity at both structural and functional levels. Imaging studies have reported associations between long‐term dance training and greater gray matter volume in regions involved in cognition and emotion regulation, including the superior temporal gyrus, cingulate cortex, and insula (Barnstaple et al. 2021; Burzynska et al. 2017; Mitterová et al. 2021). Some evidence suggests that choreographed dance may produce different or more extensive structural changes in the brain than conventional fitness training (Burzynska et al. 2017). Functional magnetic resonance imaging studies also show that dancers exhibit greater neural synchronization when observing dance, reflecting enhanced brain functional integration derived from their training experience (Rektorova et al. 2020). Dance also enhances the flexibility of brain networks related to self‐perception, engaging coordinated mechanisms across regions involved in aesthetic experience, associative processing, and body schema representation (Cross 2025; Theofanopoulou 2025). These findings are consistent with the view of dance as a multimodal activity that simultaneously engages sensory, motor, and cognitive circuits.

The integrative nature of dance may contribute to a range of clinical and functional benefits across diverse populations. Interventional evidence suggests that, in individuals at high risk for AD and those with mild cognitive impairment (MCI), a three‐month aerobic dance intervention was associated with increased hippocampal volume and improved memory performance (Zhu et al. 2022). Previous systematic reviews and meta‐analyses further support the potential benefits of dance interventions for cognitive health in older adults. Evidence suggests that dance interventions may improve global cognition and memory in older adults, although findings regarding executive function remain mixed (Meng et al. 2020). Among individuals with mild cognitive impairment, aerobic dance and other dance‐based interventions may improve global cognition, memory, visuospatial and language functions, physical function, and quality of life. However, these findings should be interpreted with caution due to heterogeneity in dance types, intervention durations, and outcome measures (Wu et al. 2021; Zhu et al. 2020). Ongoing randomized research is also examining whether genetic risk variants, such as ABCA7, may moderate the effects of cardio‐dance interventions on brain health outcomes in older adults (Gluck et al. 2023). For patients with PD, dance interventions may improve motor performance, balance, self‐esteem, and quality of life (Feenstra et al. 2022). In dementia care, dance activates memory‐related neural networks and strengthens emotional bonds with caregivers, underscoring its unique socioecological value (Blasi et al. 2025). Furthermore, dance interventions have been associated with improvements in motor control, emotional regulation, and behavioral initiative in patients with schizophrenia (Guan et al. 2023). Dance has also been investigated as a supportive intervention across mental health, addictive, and neurodevelopmental conditions. Dance interventions may support attention, motor control, and cognitive function through changes in distributed brain networks. In children with neurodevelopmental conditions, dance interventions may support motor, cognitive, and social functioning (Bégel et al. 2022; Cox and Youmans‐Jones 2023).

A defining feature of dance is the engagement of neural systems involved in social cognition. Dance requires coordinated movement and interpersonal attunement to musical rhythm, engaging the prefrontal and occipital cortices as well as the mirror neuron system. The coordinated recruitment of these regions may support empathy and social cognition (Bigand et al. 2025; Christensen et al. 2020; Theofanopoulou 2025). Moreover, participation in dance and improvisation may strengthen the sense of agency and support emotional regulation in clinical populations, including individuals with dementia (Blasi et al. 2025). This integration may also promote a psychological state of flow, characterized by deep immersion and associated with greater mindfulness, reduced stress, and enhanced subjective well‐being (Laird et al. 2021; Moyle et al. 2021).

Overall, dance extends traditional exercise by embedding physical training within rhythmic, emotional, and social contexts (Delabary et al. 2024; Theofanopoulou 2025). Through the combined impact of music and movement, dance integrates perceptual, motor, emotional, and social processes, thereby fostering a sense of belonging and self‐worth (Borowski 2021; Kaul 2024; Klyshbayev et al. 2025; Lee et al. 2020; Roaina et al. 2024; Satyal et al. 2021).

**FIGURE 2:**
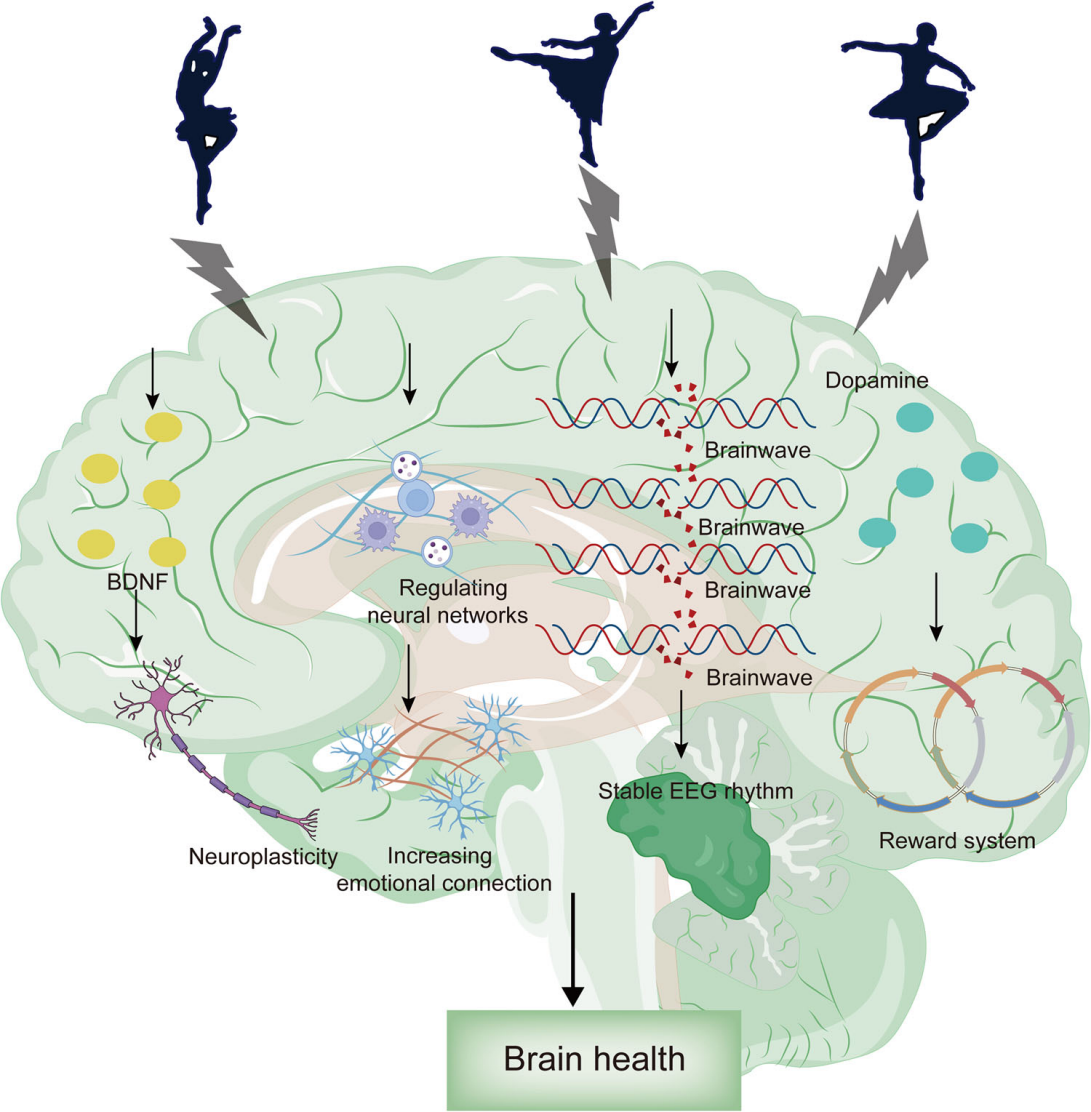
Multimodal neural mechanisms through which dance promotes brain health. Abbreviations: BDNF, brain‐derived neurotrophic factor; EEG, electroencephalogram.

These effects may arise from coordinated interactions between the brain and body. By integrating auditory, visual, motor, and socio‐emotional information, dance engages distributed neural networks involved in cognition, emotion, motor control, interoception, and social processing. Coordinated activity across these networks may upregulate BDNF, enhance synaptic plasticity, promote neural oscillatory synchronization, and recruit reward–motivation circuits, thereby supporting cognitive, emotional, and motor functions (Blasi et al. 2025; Guan et al. 2023; Qi et al. 2024; Teixeira‐Machado et al. 2019) (Figure 2).

Overall, dance represents an ecologically relevant multimodal intervention that integrates music, physical movement, cognitive challenge, emotional engagement, and social interaction. Its reported benefits may arise from the combined contributions of these components rather than from the interaction between music and exercise alone. Dance may therefore serve as a useful model for investigating integrated brain–body interventions, although the specific and interactive contributions of its individual components remain to be clarified. Current evidence suggests potential therapeutic and preventive value across populations affected by cognitive impairment, mental health conditions, addictive behaviors, and neurodevelopmental disorders.

## 7 Limitations and Future Directions

Despite the wealth of evidence examining the positive effects of music and exercise on brain health across behavioral, electrophysiological, neuroimaging, and molecular levels, research on their combined interventions remains in its infancy. While active music combined with exercise has been applied in rehabilitation research, several limitations persist (Schneider et al. 2022a). First, the number of empirical studies is limited, particularly large‐sample, long‐term randomized controlled trials, making it difficult to fully verify efficacy in neurological disorders such as cognitive impairment, depression, and PD. Second, existing studies mainly address behavioral or symptomatic improvements, with insufficient direct evidence regarding neurobiological mechanisms. Most remain at the level of indirect speculation about their respective mechanisms and lack systematic integration of “synergistic signal pathways.” Importantly, most existing studies have not used full factorial designs that separately compare music alone, exercise alone, their combination, and an appropriate control condition. Consequently, current evidence cannot determine whether the observed combined effects are simply additive or statistically super‐additive; future studies should employ factorial designs and formal interaction analyses to distinguish these possibilities. Although sports psychology research has examined how exercise type and intensity interact with music to affect attention, emotion, and psychophysiological responses, further exploration is highly desired (Karageorghis et al. 2018).

Furthermore, substantial heterogeneity in intervention protocols limits the consistency and comparability of findings. Variations in music type, tempo, volume, and degree of personalization, as well as exercise mode, intensity, frequency, and duration, may affect the reproducibility of intervention outcomes (Fan et al. 2024; Lu et al. 2024). For example, the effects of music during acute exercise on executive functions and affective responses appear to be context‐dependent, with exercise intensity and participant age contributing to between‐study variability, while substantial residual heterogeneity remains (Danso et al. 2025). Moreover, the field currently lacks a standardized framework for defining and reporting the dose of combined music–exercise interventions. Future studies should therefore report music characteristics, including music type, tempo, volume, and degree of personalization; exercise prescription, including mode, intensity, frequency, and duration; and session structure, including the timing, sequencing, and synchronization of music and movement. Such standardized reporting would improve the comparability, reproducibility, and clinical translation of future studies. Future studies should also distinguish between different forms of music engagement, including passive listening, singing, instrumental performance, rhythmic auditory cueing, and dance, because these interventions may differ in their motor, cognitive, emotional, and social components. Individual differences have also received insufficient attention. For example, the moderating effects of age, sex, baseline cognitive status, and genotype on intervention responses have not been systematically evaluated (Ballmann 2021; Suwabe and Kawase 2025; Zhang et al. 2024). Dance is a complex multimodal intervention that includes music, physical movement, cognitive demands, and social interaction. Accordingly, existing studies cannot clearly isolate the specific contributions of music and exercise, highlighting the need for better‐matched comparison designs.

Future research should advance in several directions: (1) Conducting multicenter, cross‐population, large‐sample clinical trials integrating standardized protocols with multimodal technologies such as functional imaging and metabolomics to elucidate the neural basis of music–exercise synergy; (2) Developing personalized music‐exercise interventions by integrating biomarkers, genetic profiles, and lifestyle factors to optimize precision matching; (3) Strengthening long‐term follow‐up and translational studies to assess sustainability and accessibility in rehabilitation and community health; (4) Promoting mechanism‐oriented basic research using animal models and brain organoids to dissect molecular signal pathways in neural plasticity, energy metabolism, and inflammation, thereby laying a stronger theoretical foundation for clinical application.

## 8 Conclusion

**FIGURE 3:**
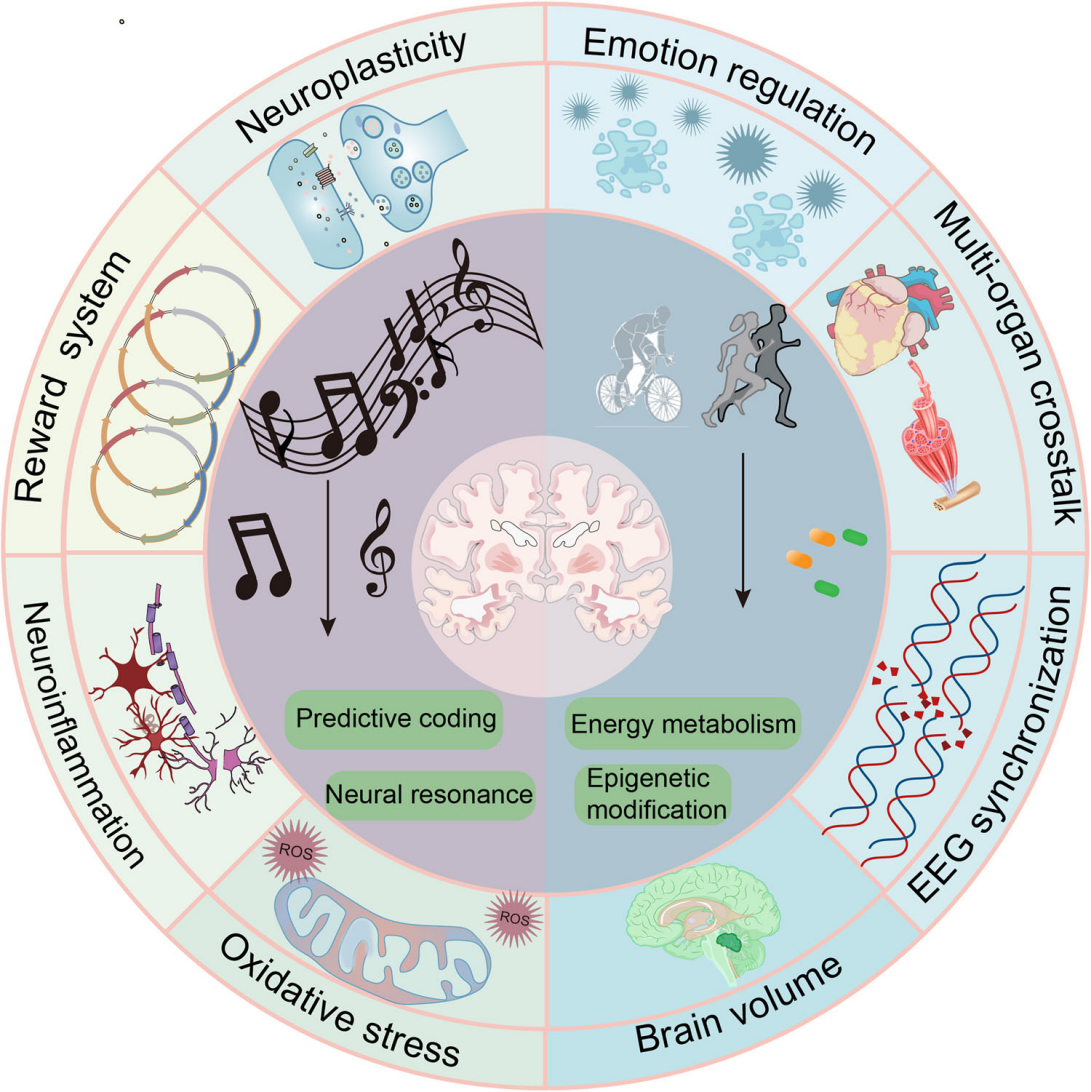
Synergistic mechanisms of combining music and exercise interventions on brain health. Music and exercise engage complementary neural and systemic pathways that converge on reward signaling, neuroplasticity, emotional regulation, neural oscillations, and the regulation of inflammation and oxidative stress. Through these shared and interacting mechanisms, combined music–exercise interventions may support cognitive, emotional, and overall brain health.

Music and exercise, as widely accessible non‐pharmacological interventions, confer substantial and pleiotropic benefits for brain health, including neuroplasticity, neurotransmitter regulation, energy metabolism, and inflammation control. Their combined application may provide additional benefits for cognitive and emotional outcomes, although current evidence is insufficient to determine whether these effects are additive or genuinely super‐additive. As a paradigm of music–movement integration, dance can activate extensive brain networks through multimodal integration of rhythm, movement, and emotion, enhance cognitive and emotional regulation, and demonstrate unique value in clinical and rehabilitation practice. In summary, music and exercise may interact across multiple levels through convergent neural and systemic mechanisms that support brain health. Dance provides a vivid paradigm of this integrated approach and opens new avenues for developing personalized, comprehensive strategies to advance brain health (Figure 3). Given their safety, accessibility, and broad benefits, integrated music–exercise protocols, exemplified by dance, may serve as promising therapeutic tools. They may also provide viable public health strategies for promoting brain health across diverse community populations.

## Author Contributions

**Sanxia Yang**: conceptualization, writing – original draft preparation, funding acquisition, writing – review and editing, supervision. **Qingluan Liu**: conceptualization, writing – original draft preparation. **Minghui Wang**: conceptualization, writing – original draft preparation, visualization. **Yidan Zhang**: visualization.

## Funding

This research was funded by the Philosophy and Social Science Research Key Project of the Hubei Provincial Department of Education (No. 23D039).

## Conflicts of Interest

The authors declare no conflicts of interest.

## Data Availability

No datasets were used for the research described in the article.
